# Growth dynamics among adolescent girls in Bangladesh: Evidence from nationally representative data spanning 2011–2014

**DOI:** 10.1371/journal.pone.0255273

**Published:** 2021-07-29

**Authors:** A. M. Adams, A. Khan, A. S. Roy, Md. T. Hassan, M. K. Mridha, N. U. Ahmed, P. Mustaphi, I. Chowdhury, R. Khondker, Z. Hyder

**Affiliations:** 1 Department of Family Medicine, Faculty of Medicine and Health Sciences, McGill University, Montreal, QC, Canada; 2 BRAC James P Grant School of Public Health, BRAC University, Dhaka, Bangladesh; 3 Department of Economics, Uppsala University, Uppsala, Sweden; 4 Shornokishori Network Foundation, Dhaka, Bangladesh; 5 UNICEF Bangladesh Country Office, Dhaka, Bangladesh; 6 Global Alliance for Improved Nutrition (GAIN), Dhaka, Bangladesh; 7 The World Bank Group Cambodia, Phnom Penh, Cambodia; University of Western Australia, AUSTRALIA

## Abstract

**Background:**

Adolescence is the last opportunity to reverse any growth faltering accumulated from fetal life through childhood and it is considered a crucial period to optimize human development. In Bangladesh, a growing double burden of underweight and obesity in adolescents is recognized, yet limited data exists on how, when, and where to intervene. This study assesses the dynamics of growth among adolescent girls in Bangladesh, providing insight about critical junctures where faltering occurs and where immediate interventions are warranted.

**Methods:**

We pooled data from Bangladesh’s Food Security and Nutrition Surveillance Project collected between 2011 and 2014 to document the age dynamics of weight and linear growth. 20,572 adolescent girls were measured for height and 19,345 for weight. We constructed growth curves for height, weight, stunting, and underweight. We also stratified growth dynamics by wealth quintile to assess socioeconomic inequities in adolescent trajectories.

**Results:**

Height-for-age z-score (HAZ) in Bangladeshi girls deteriorates throughout adolescence and especially during the early years. Mean HAZ decreases by 0.20 standard deviations (sd) per year in early adolescence (10–14 years) vs 0.06 sd/year during late adolescence (15–19 years), while stunting increases by 16 percentage points (pp) vs 6.7 pp, respectively. Conversely, BMI-for-age z-score (BAZ) increases by 0.13 sd/year in early adolescence vs 0.02 sd/year in late adolescence, and underweight decreases by 12.8 pp vs 3.2 pp. Adolescent girls in all socioeconomic groups show a similar pattern of HAZ and BAZ dynamics, but the curve for the richest quintile stays above that of the poorest across all ages.

**Conclusions:**

Trends and levels of stunting and underweight among adolescent girls in Bangladesh are worrisome, suggesting substantial linear growth faltering in early adolescence, with improving weight-for-age occurring only as linear growth slows and stops. Given the rising burden of non-communicable diseases (NCDs) in Bangladesh and emerging evidence of the link between stunting and later chronic diseases, greater attention to adolescent growth and development is needed. Our findings suggest that, to address stunting, interventions in early adolescence would have the greatest benefits. School-based interventions could be a way to target this population.

## Introduction

Adolescence is the second most crucial period of human growth and development where rapid changes in body size, composition, physiology, and endocrine systems occur simultaneously. Adolescence is also widely considered the last opportunity to reverse prior growth faltering due to undernutrition, infection, and environmental stress accumulated from fetal life through childhood [1–4].

Adequate nutrition during adolescence is important given its influence on hormonal mechanisms related to growth and puberty [5]. For instance, low weight-for-height can have serious long-term effects such as inadequate bone mineralization leading to osteoporosis [6], and increased morbidity and mortality from infections. On the other hand, stunting or low height-for-age in adolescence is associated with overweight later in life, with a higher prevalence of obesity (body mass index (BMI) >23) observed in stunted individuals [7] due to lower metabolic efficiency and resting energy expenditure, and a higher tendency to store fat [8, 9]. Evidence further suggests that these processes may lead to higher long-term susceptibility to non-communicable diseases (NCDs) [7, 10]. For example, evidence from the Dutch Famine found a significant association between exposure to undernutrition during adolescence and the risk of peripheral artery disease and diabetes among females later in life [11]. A similar study in China showed a link between severe underweight in adolescence and dyslipidemia in adulthood [12].

The impacts of poor adolescent nutrition can also have intergenerational effects. For girls, stunting before pregnancy is a risk factor for poor fetal growth (leading to small-for-gestational-age and low-birth-weight babies) as well as perinatal risks such as still- and preterm-births [4, 13, 14]. Undernutrition early in life can result in deficiencies in muscular strength and working capacity, or compromised intellectual development, school attendance, academic performance, and social skills [15].

Assuming that a country’s economic growth depends on having a healthy cohort of young people becoming economically and socially productive adults [16], investments in adolescent nutrition are imperative. This is particularly important for the many low- and middle-income countries (LMICs) to optimize the so-called ‘demographic dividend’ [17]. However, health and nutrition data on adolescents are limited in these contexts [18], and nutrition programming for this population is often not prioritized. Little is known, for instance, on the age dynamics of height and weight over adolescence. When does growth faltering occur (if at all)? Do height and weight follow different time-paths? Do growth dynamics differ across the socioeconomic spectrum, between urban, rural and in hard-to-reach areas, in school going and non-school going adolescents, and/or in married or unmarried adolescents? Evidence on these issues is critical for the optimal design and timing of potential remedial or preventative interventions. Part of the current knowledge gap on adolescent nutrition is a function of the disproportionate focus on the first 1,000 days of life, when the foundations of health, growth, and neurodevelopment across the lifespan are established [18]. It also points to insufficient recognition of the long-term risks of adolescent undernutrition, and their health and societal impacts [19].

In Bangladesh, these risks are accentuated among girls, where ages of marriage and childbearing are among the lowest in the world [20], and rates of stunting and underweight are among the highest in the region. According to cut-off points where mothers under 45 kg and under 145 cm are deemed at obstetric risk, a study found that this represented 83% and 23% of 16-year-old Bangladeshi girls, respectively [21]. Moreover, since undernutrition results in delayed onset of the growth spurt, and early pregnancy is common, the growth of pregnant Bangladeshi girls may be prematurely arrested [22].

Bangladesh’s National Plan of Action for Adolescent Health Strategy 2017–2030 recognizes the crisis of malnutrition among adolescents, and the growing double burden of undernutrition and overweight and obesity. Lacking, however, is evidence-based insight on when and where to intervene. In this study we explore the dynamics of growth among Bangladeshi girls over the adolescent period, with a view to identifying critical moments where faltering occurs, and when interventions might best be directed.

## Methods

Data for this study are furnished by the Bangladesh Food Security and Nutrition Surveillance Project (FSNSP) covering the period 2011 to 2014. FSNSP represents a unique source of nationally representative data on food security and nutrition based on surveys of more than 25,000 households per year. From each surveyed household, one non-pregnant woman or adolescent girl (10–49 years of age) is randomly selected for anthropometric measurements of height, weight, and mid-upper arm-circumference (MUAC), providing a sample of over 4,500 adolescent girls (aged 10–19 years) per year. Details on sampling, anthropometry and ethical procedures are published elsewhere [23–26]. In total, A separate questionnaire was administered at the household level to ascertain rural/urban location, household composition, socioeconomic status, and food security.

Because the survey was not following the same individuals over time, we constructed growth curves by pooling data from repeated cross-sections surveyed over 4 years, yielding a final sample of 20,572 adolescent girls with height measurements, and 19,345 adolescent girls with weight measurements. We stratified the pooled sample into 10 age-groups (1 per year of age, 10–19) and estimated mean anthropometric indices for each group. Each of these values was plotted against age to create a growth curve. There are several concerns in using this approach. First, growth dynamics might have changed between 2011 and 2014, the start and endpoint of the survey period. To check for this potential bias, we constructed separate growth profiles using data from the years 2011 and 2014, but found no notable differences in the shape of the curve (Fig 2 for an analysis using HAZ scores).

A second concern is that a comparison of height of 10 and 13-year-old girls in a cross-section, for example, may also capture underlying secular trends: i.e. 13-year-old girls may be shorter for their age vis-à-vis their younger counterparts due to growth faltering as well as secular improvements in population height over time. To verify, we estimated cohort-specific growth curves by constructing age-differentiated cohorts in 2011 and followed them for 3 years. The ‘synthetic cohort’ strategy exploits the fact that adolescents were randomly sampled across consecutive years, and thus, were statistically similar for a given cohort over time. To illustrate, we constructed a synthetic cohort for those aged 11 years in 2011 by using samples of girls aged 12 in 2012, 13 in 2013, and 14 in 2014. This analytical approach has been employed in similar settings where longitudinal data at the individual level is not available [27]. Results from this exercise (see S1 Fig for details) suggest that our comparisons using the pooled sample are not driven by secular trends. As a further check, we compared our estimates with findings from the IFPRI Bangladesh Integrated Household Panel Survey (2011–12 and 2015) which provides two data points for each individual in the survey. Once again, reasonable correspondence was noted (see S2 Fig for details); [28, 29]. Therefore, to maximize sample size, we use the pooled sample for all reported analyses.

We present growth curves for the following seven measures of nutritional status: height (in centimeters); height-for-age z-scores or HAZ, which represents a measure of chronic undernutrition [30]; stunting, which measures the degree of chronic undernutrition with reference to a cut-off of HAZ<-2 (moderately or severely short for age); weight (in kilograms); body mass index (BMI; in kilograms of weight per square meter in height); BMI-for-age z-scores or BAZ, which captures acute undernutrition [30]; and underweight, which measures the degree of acute undernutrition with reference to a cut-off of BAZ <-2 (moderately or severely thin). We use local polynomial smoothing to enable easy visualization of dynamics [31]. Furthermore, we stratify growth dynamics by wealth quintiles to assess socioeconomic inequities in the adolescent growth trajectory.

We also report changes in outcomes in both absolute and relative terms, stratifying by early (10–14 years) and late (15–19 years) adolescence. For instance, to analyse changes in height (cm) during early adolescence we estimated the following: absolute change = height(14)-height(10); average annual absolute change = average of annual or year-to-year absolute changes between ages 10 and 14 years; relative change = 100*(height(14)/height(10) - 1); and average annual relative change = average of annual or year-to-year relative changes between ages 10 and 14 years.

## Results

### Dynamics in height

**Fig 1:**
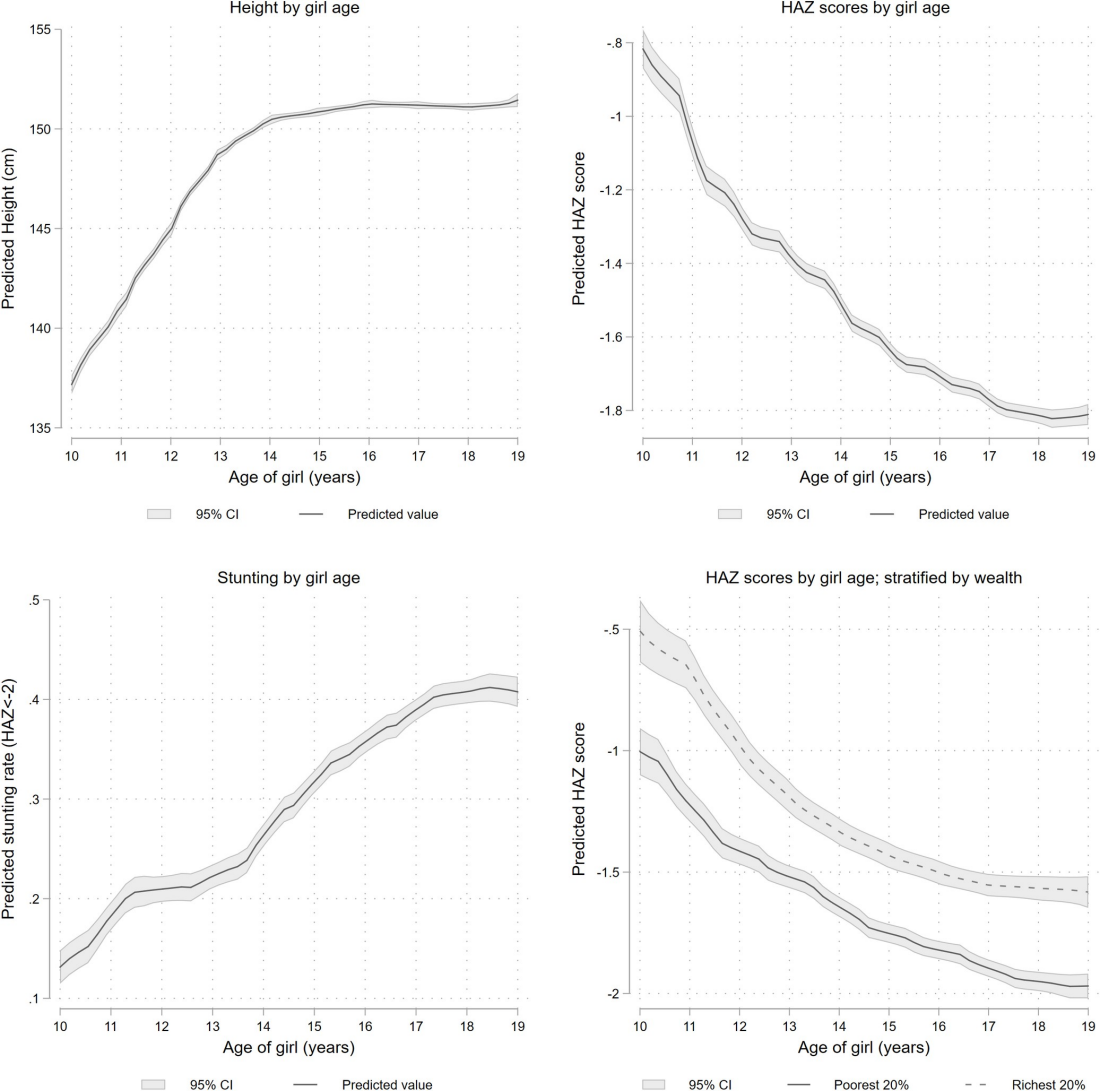
Age dynamics in linear growth of adolescent girls in Bangladesh. (A) Height by girl age (B) HAZ scores by girl age. Z-scores express height in terms of standard deviations below or above the reference median value. (C) Stunting by girl age. (D) HAZ scores by girl age, stratified by wealth.

Fig 1A and 1B report mean height and height-for-age z-score by age, respectively. In addition, Table 1 reports the corresponding estimates of absolute and relative changes during early (10–14 years) and late (15–19 years) adolescence, while S1 Table presents estimates using sampling weights. There is a linear increase in height (cm) in early adolescence, somewhat tapering off at the latter years (Fig 1A). In the early adolescent period a gain of 13.3 cm (9.71%) is apparent, with a mean annual gain of 3.33 cm. This decreases to 0.60 cm (0.4%) during late adolescence with a mean growth of 0.19 cm/year (Columns 1 through 3 in Table 1).

**Table 1:** Changes in height, height-for-age z-score, and stunting during early and late adolescence.

	Height (cm)				Height-for-age z-score				Stunting (HAZ<-2)			
Period	(1)	(2)	(3)	(4)	(5)	(6)	(7)	(8)	(9)	(10)	(11)	(12)
	Absolute change (cm)	Average annual absolute change (cm)	Relative change (%)	Average annual relative change (%)	Absolute change	Average annual absolute change	Relative change (%)	Average annual relative change (%)	Absolute change (pp)	Average annual absolute change (pp)	Relative change (%)	Average annual relative change (%)
Early Adolescence (10–14)	13,31	3,33	9,71	2,34	-0,79	-0,20	-112,87	-22,03	16,06	4,01	172,87	34,61
Late Adolescence (15–19)	0,60	0,19	0,40	0,13	-0,13	-0,06	-8,13	-3,70	6,72	2,77	20,69	9,65

Height-for-age z-score (hereafter, HAZ), which captures rates of chronic nutrition with reference to a healthy norm, declines throughout adolescence, with a much sharper fall during the early years (Fig 1B). In fact, average HAZ decreases by over 100% during early adolescence compared to an 8.13% decline during the later adolescent years (Column 7 in Table 1). Over the entire period, HAZ falls from -0.7 to -1.8 standard deviations (sd), meaning that girls’ height is on average 0.7 sd below the reference median at age 10 years, followed by sustained growth faltering over adolescence to reach a mean height of 1.8 sd below the reference at 19 years of age (Fig 1B).

Correspondingly, during early adolescence, levels of stunting rise by a remarkable 173% compared to 20% in late adolescence (Column 11 in Table 1; Fig 1C). This represents an absolute increase of 16 percentage points (pp) in the early adolescent period for a mean increase of 4 pp/year, and a 6.7 pp increase during late adolescence for a mean increase of 2.77 pp/year (Columns 9 and 10 in Table 1).

On average, adolescent girls from the richest 20% of households are taller than their poorest counterparts (by ~0.5 HAZ). However, as observed in Fig 1D, similar growth dynamics are apparent across the adolescent period irrespective of wealth quintile. The wealth gap persists, but the trend line runs in parallel.

**Fig 2:**
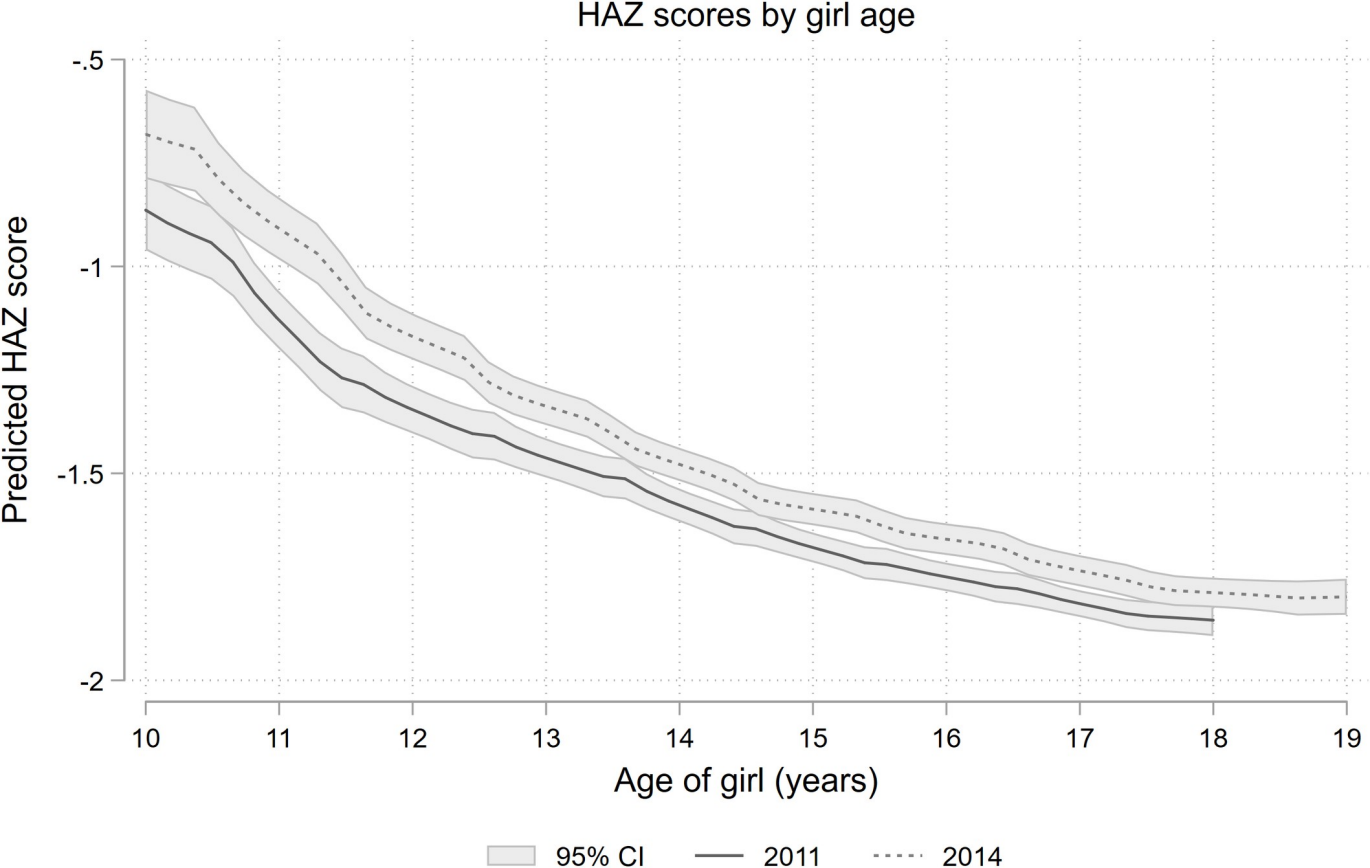
HAZ scores by girl age– 2011 vs 2014.

Finally, Fig 2 plots HAZ growth curves in 2011 and 2014, indicating a slight improvement in HAZ in 2014 compared to curves constructed for 2011.

### Dynamics in weight

**Fig 3:**
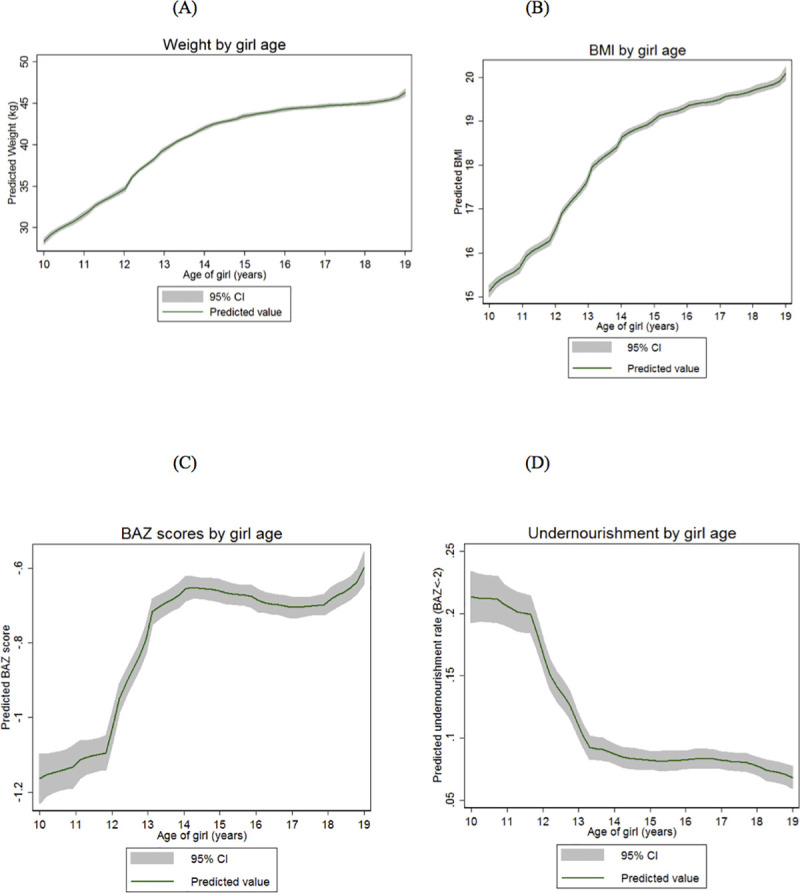
Age dynamics in weight of adolescent girls in Bangladesh. (A) Weight by girl age. (B) BMI by girl age. (C) BAZ scores by girl age. Z-scores express BMI as a number of standard deviations below or above the reference median value. (D) Undernourishment by girl age. Undernourishment is defined as BAZ <-2.

Fig 3A and 3B report average weight and BMI by age, respectively, and Table 2 presents corresponding estimates of absolute and relative changes over the early (10–14 years) and late (15–19 years) adolescent period. S2 Table calculates the same estimates using sampling weights. Similar to height gain trajectories, weight rises linearly during early adolescence at a mean increase of 3.44 kg/year, compared to 0.84 kg/year in the older adolescent period (Column 2 in Table 2; Fig 3A). Most weight gain occurs during early adolescence, with approximately 15 kg added between 10 and 14 years, compared to a 3 kg increase from 15 to 18 years. Indeed, weight almost stabilizes (the curve flattens) between the ages of 16 and 18. Similar trends are observed for BMI (Fig 3B).

**Table 2:** Changes in weight, BMI-for-age z-score, and underweight during early and late adolescence.

	Weight (kg)				BMI-for-age z-score				Underweight (BAZ<-2)			
Period	(1)	(2)	(3)	(4)	(5)	(6)	(7)	(8)	(9)	(10)	(11)	(12)
	Absolute change (kg)	Average annual absolute change (kg)	Relative change (%)	Average annual relative change (%)	Absolute change	Average annual absolute change	Relative change (%)	Average annual relative change (%)	Absolute change (pp)	Average annual absolute change (pp)	Relative change (%)	Average annual relative change (%)
Early Adolescence (10–14)	13,75	3,44	48,48	10,41	0,52	0,13	44,72	12,98	-12,79	-3,20	-59,38	-17,50
Late Adolescence (15–19)	2,89	0,84	6,64	1,92	0,10	0,02	15,77	2,56	-2,31	-0,61	-28,75	-7,48

Comparing Tables 1 and 2, weight appears to increase at a greater rate with age than height. For instance, during early adolescence, the relative gain in average weight is close to 50% compared to a 10% gain in height (Column 3 in Tables 1 and 2). Consequently, in sharp contrast to the secular decline in HAZ observed earlier, BMI-for-age z-score (hereafter, BAZ) registers a steep rise in early adolescence, stabilizing thereafter (Fig 3C). A corresponding fall in the rate of undernourishment (BAZ<-2) by 12.8 pp is also noted during the early adolescent years (Column 9 in Table 2; Fig 3D). The prevalence of undernourishment more than halves during this period, declining from over 20 pp to under 10 pp (Column 11 in Table 2; Fig 3D).

**Fig 4:**
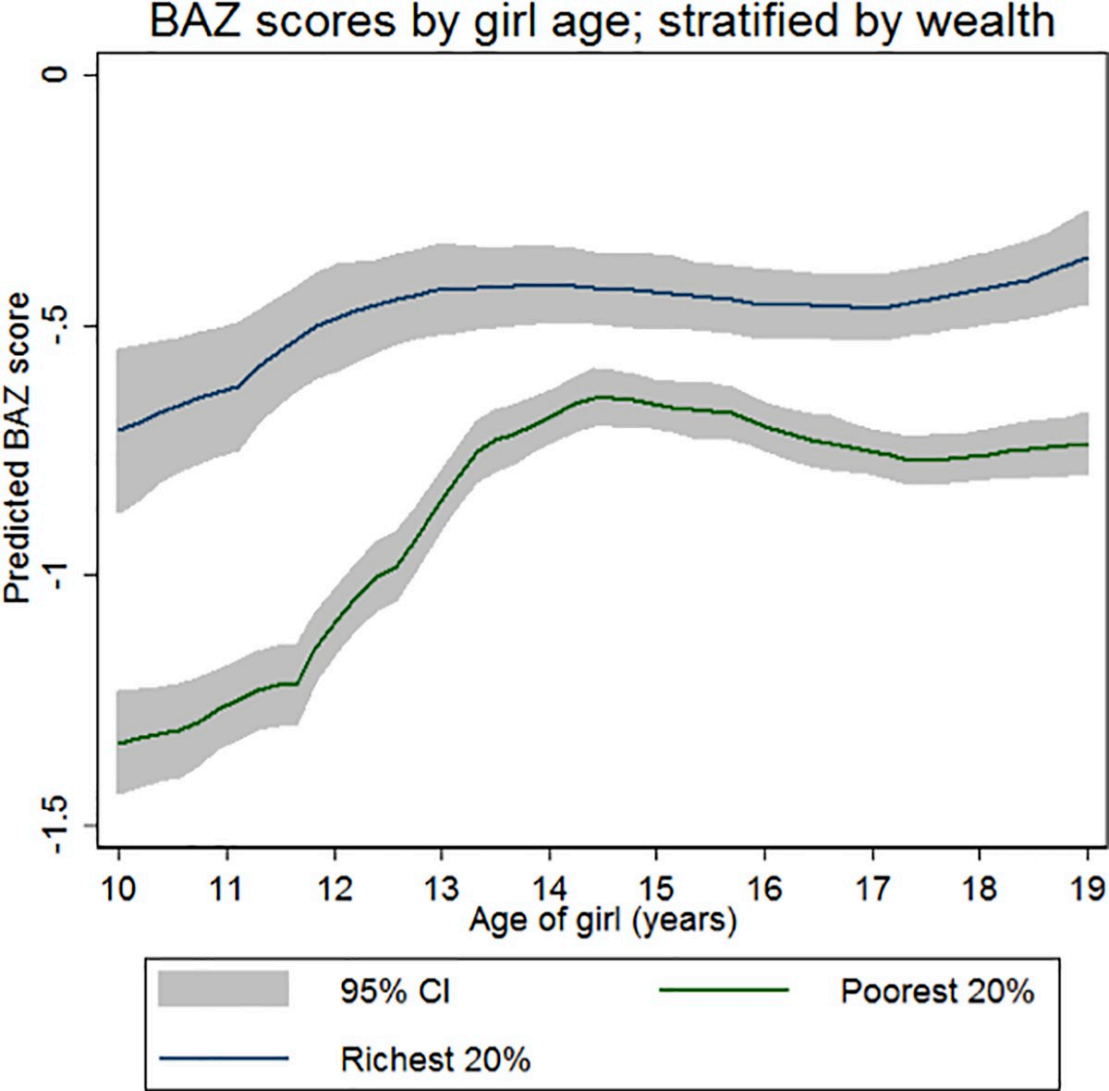
BAZ scores by girl age, stratified by wealth.

Fig 4 shows a persistent socioeconomic gap in BAZ comparing girls from poorest and richest wealth quintile households across the adolescent period. However, unlike HAZ, there is some evidence of catch-up vis-à-vis reference median values, during the early years.

## Discussion

The high rate of adolescent undernutrition in our sample is consistent with global data identifying South Asia and Bangladesh as places where rates of underweight and stunting are well above the healthy norm [4]. Our findings revealed a rise in stunting (Fig 1C) over the adolescent period, and a decline in underweight prevalence (Fig 3D) as linear growth stagnates and stops in late adolescence. Similar trends for adolescent growth are observed in the region–including in India, Nepal, Thailand, and Indonesia, with a rise in stunting as girls move into late adolescence, and higher rates of underweight in the early adolescent period [32–36].

Looking at our results more closely, some improvements in overall levels of stunting are apparent comparing data from 2011 and 2014, although age dynamics remains the same (Fig 2). In both years, HAZ score deteriorates over the adolescent period, with the biggest drop occurring between 10 and 12 years of age. While girls still have HAZ scores well below the healthy norm in 2014, they are less stunted on average than in 2011. A similar downwards trend in rates of stunting among Bangladeshi adolescents has been observed elsewhere [37]. Among the potential factors driving improvements in growth are the integration of nutrition-specific interventions into national primary healthcare services in 2011 [38], improved sanitation, antenatal care access, increased parental schooling, and reductions in poverty and fertility rate [31].

According to Headey [31], the factor with the strongest contribution to changes in HAZ among under-five children is asset ownership. Our study corroborates the negative association between relative wealth and stunting among the adolescent age group. When HAZ scores are stratified by wealth, growth curves for richest and poorest quintiles have a similar shape (Fig 1D). Although the curve for the richest quintile remains above the curve for the poorest at every age, even girls in this group have suboptimal HAZ scores. In fact, there is evidence that dietary diversity among adolescent girls in our sample declined significantly over the period 2011 to 2014 [39].

Also of concern is the rapid rate of weight gain across the adolescent period especially among older girls (Fig 3D). A study in Philippines found that girls had an increased odds of high blood pressure if they had low birth BMI and high adolescent BMI (compared with those with low BMI in birth and adolescence, or those with high BMI in birth and adolescence) [40]. Further, the odds of high blood pressure in girls were greater in those experiencing larger weight increments between ages 8 to 16 years [40]. Another study noted that rapid weight gain among individuals born small at birth, particularly in early childhood, increases risk of chronic disease in later life [14]. Both studies suggest that observed trends in adolescent BMI may heighten risks of overweight, obesity and chronic disease in adulthood, and in the context of Bangladesh, will further augment a growing prevalence and incidence of metabolic disorders in women [41].

### Policy implications

Together, our findings highlight the importance of taking advantage of the ‘second window of opportunity’ by bolstering the nutrition of girls as they enter adulthood. Specifically, we suggest that interventions targeting early adolescence should be prioritized as this is the period where rates of stunting increase most rapidly, and when interventions might have the highest returns. Starting into the late adolescent period, growth levels off, leaving girls at a subnormal height while weight gain continues. The need for early intervention is even more urgent in the context of Bangladesh, given the known risks of stunting and overweight to obstetric outcomes, and the high prevalence of child marriage [42] and adolescent pregnancy (113/1000 among 15–19 year olds) [43].

On a positive note, high rates of lower-secondary attendance among girls in Bangladesh–over 90% in 2019 –provide a vital opportunity for action on girl’s nutrition. School-based nutrition programs including tailor-made supplementation and education interventions are particularly promising [44]. The strong relationship between poverty and girls’ undernutrition also merits attention given its pervasive effects on growth, and later adolescent [45] and adult life [46]. Here investments in food security, access to health services, and water and sanitation are crucial, even more so given their proximal impacts on dietary adequacy, breastfeeding, and management of infectious diseases, all of which affect physical growth. Adolescent Friendly Health Corners (AFHCs), run by the Directorate General of Family Planning (DGFP), offer another strategic entry point. Taking advantage of this existing sub-district level platform, current sexual and reproductive health services might be broadened to include adolescent-focused nutrition education and support.

Potential cost-effective interventions have been identified in a recent cost-benefit study of interventions to support investments in adolescent nutrition [39]. Findings identified secondary schools as effective delivery platforms for adolescent nutrition interventions such as nutrition fairs, nutrition promotion through school clubs, school health checks, and initiatives to increase physical activity. The study also recommended extending nutritious school meals provision in secondary schools, and integrating deworming and menstrual hygiene management in school-based hygiene and sanitation programs [39]. Other actions included scaling-up existing government investments in adolescent nutrition; the addition of recommended interventions into the national budget and 5-year plan (including at least one adolescent nutrition indicator to measure progress), and prioritizing actions to delay the first pregnancy of married adolescents. Considering that funding is already available under the Second National Plan of Action on Nutrition II (NPAN2) for different Ministries, these actions are possible and should target areas of the country where performance on nutrition indicators is poorest.

### Limitations

Pooling nationally representative data from repeated cross-sectional surveys over a four-year period, this study provides novel descriptive evidence on the growth dynamics of adolescent girls in Bangladesh. However, future measurement exercises should follow a longitudinal approach (i.e. surveying the same sample of girls over time) to address potential concerns posed by underlying secular trends, and to better specify key factors driving changes in nutritional outcomes over the adolescent period. The lack of data for adolescent boys is another limitation. Given the importance of nutrition to health in adulthood, sex and gender disaggregated data are critical for optimal policy design in countries like Bangladesh in which the health and nutritional consequences of inequitable gender norms are persistent concerns.

## Conclusions

This study assessed the dynamics of growth among adolescent girls in Bangladesh, providing insight about critical junctures where faltering occurs. Our findings reveal an increase in stunting over adolescence which is especially rapid in the early adolescent period, followed by a rise in BMI as linear growth falters, and weight increases. These findings lead to two important conclusions for policy-makers– 1) that early adolescence is a crucial period of intervention to reduce the burden of stunting, and 2) that efforts to address rising overweight in Bangladesh must engage girls entering later adolescence.

Currently, 67% of all deaths in Bangladesh are attributed to NCDs [47]. While some of this burden relates to the larger forces of globalization and the nutrition transition, stunting and underweight in childhood and adolescence are known determinants of NCD risk in later life. Bangladesh’s Second National Plan of Action for Nutrition (NPAN2) for 2015–2024 recognizes the prevalence of adolescent undernutrition, and the negative health consequences of rising adult overweight and obesity in a context of declining physical activity and low dietary adequacy [48]. Adolescent girls are specifically identified as one of the target groups for the NPAN2, which sets an indicator for 2025 to reduce malnutrition (BMI<18.5) among adolescent girls 15–19 years-old to less than 15% [49]. Nutrition interventions such as childhood nutrition education, primary school-based feeding programs, and micronutrient supplementation during pregnancy are in place. Yet, the critical “second window” of adolescent growth and development is insufficiently acknowledged and prioritized despite its link with intergenerational malnutrition and chronic disease in adulthood. The 2017–2030 National Strategy for Adolescent Health provides an opportunity to optimize this critical period of physical and behavioral development. Obvious first steps are extending school-based nutrition programing for younger adolescent girls, and as well as policies that maximize conditions for healthy adolescent growth and development including the alleviation of household poverty and child marriage.

## Supporting information

S1 Fig HAZ scores by adolescent girl age following cohorts from 2011. (TIF)

S2 Fig Age dynamics in height-for-age z-scores of adolescents; comparing FSNSP and BIHS estimates. (TIF)

S3 Fig Urban-rural differences in the growth dynamics of adolescent girls in Bangladesh. (TIF)

S1 Table Changes in height, height-for-age z-score, and stunting during early and late adolescence (using sampling weights). (DOCX)

S2 Table Changes in weight, BMI-for-age z-score, and underweight during early and late adolescence (using sampling weights). (DOCX)
