# Supplementary figures and images for: Growth dynamics among adolescent girls in Bangladesh: Evidence from nationally representative data spanning 2011–2014
Source: PLoS One. 2021 Jul 29;16(7):e0255273. doi: 10.1371/journal.pone.0255273 (PMC8321121; doi:10.1371/journal.pone.0255273)

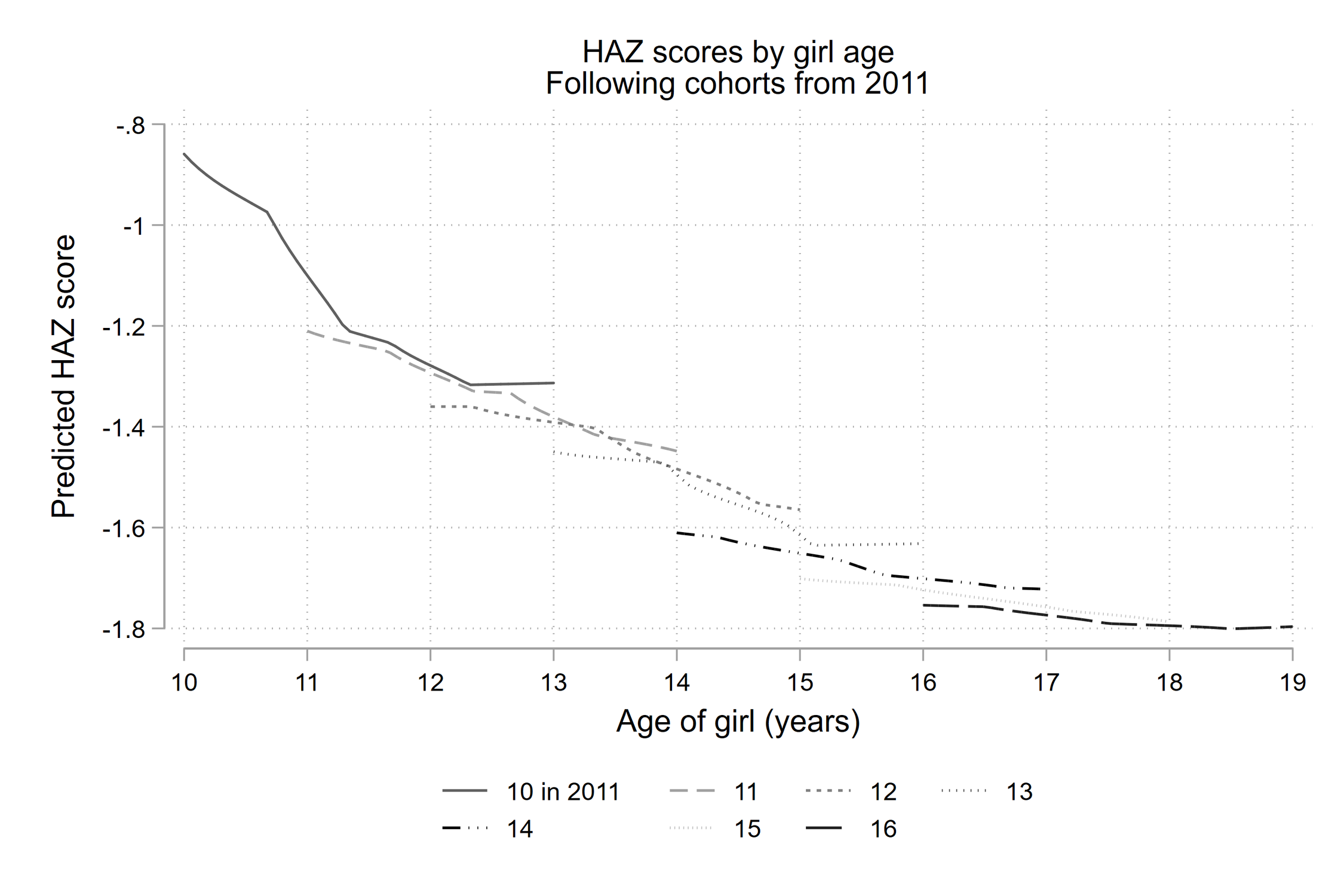

Supplement: S1 Fig — (TIF) [file pone.0255273.s001.tif]

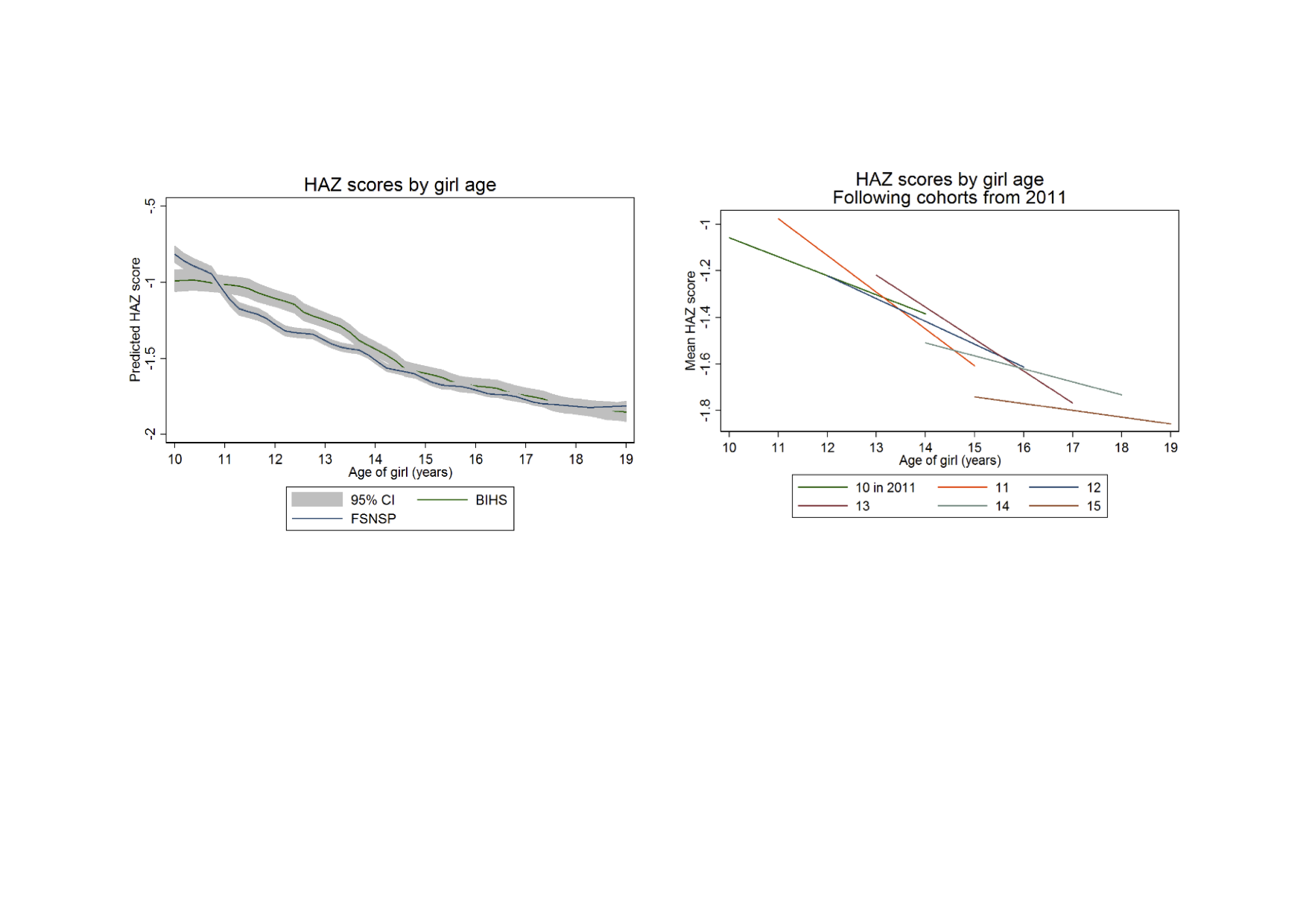

Supplement: S2 Fig — (TIF) [file pone.0255273.s002.tif]

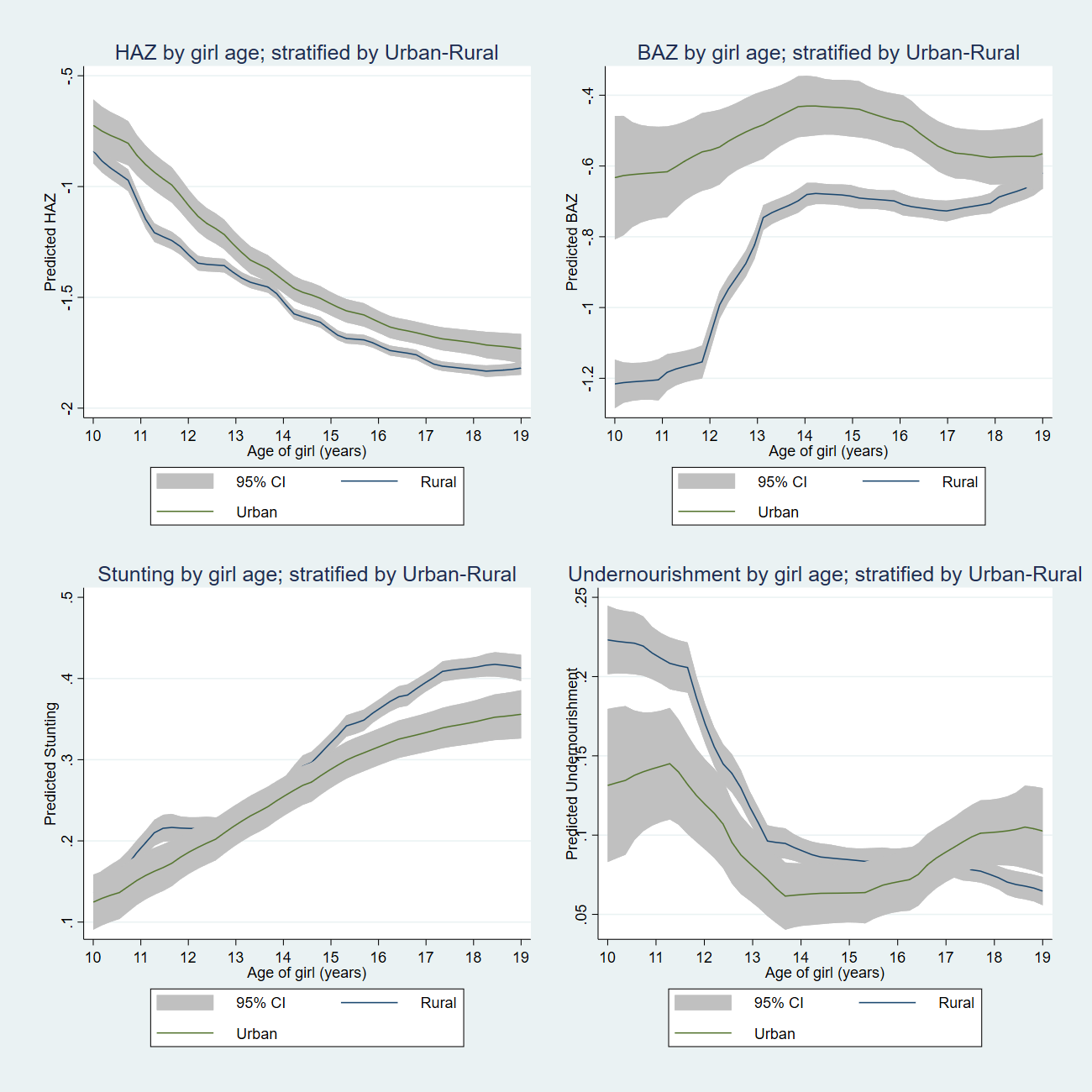

Supplement: S3 Fig — (TIF) [file pone.0255273.s003.tif]
