# Supplementary material for: Growth dynamics among adolescent girls in Bangladesh: Evidence from nationally representative data spanning 2011–2014
Source: PLoS One. 2021 Jul 29;16(7):e0255273. doi: 10.1371/journal.pone.0255273 (PMC8321121; doi:10.1371/journal.pone.0255273)
Supplement: S1 Table — (DOCX) [file pone.0255273.s004.docx]

|  | Height (cm) | | | | Height-for-age z-score | | | | Stunting (HAZ<-2) | | | |
| --- | --- | --- | --- | --- | --- | --- | --- | --- | --- | --- | --- | --- |
| Period | (1) | (2) | (3) | (4) | (5) | (6) | (7) | (8) | (9) | (10) | (11) | (12) |
|  | Absolute change (cm) | Average annual absolute change (cm) | Relative change (%) | Average annual relative change (%) | Absolute change | Average annual absolute change | Relative change (%) | Average annual relative change (%) | Absolute change (pp) | Average annual absolute change (pp) | Relative change (%) | Average annual relative change (%) |
| Early Adolescence (10-14) | 13,10 | 3,28 | 9,53 | 2,30 | -0,82 | -0,21 | -124,61 | -23,62 | 15,46 | 3,87 | 177,92 | 32,78 |
| Late Adolescence (15-19) | 0,01 | 0,15 | 0,01 | 0,10 | -0,22 | -0,07 | -13,87 | -4,07 | 7,77 | 3,10 | 24,38 | 10,92 |
