# Supplementary material for: Growth dynamics among adolescent girls in Bangladesh: Evidence from nationally representative data spanning 2011–2014
Source: PLoS One. 2021 Jul 29;16(7):e0255273. doi: 10.1371/journal.pone.0255273 (PMC8321121; doi:10.1371/journal.pone.0255273)
Supplement: S2 Table — (DOCX) [file pone.0255273.s005.docx]

|  | Weight (kg) | | | | BMI-for-age z-score | | | | Underweight (BAZ<-2) | | | |
| --- | --- | --- | --- | --- | --- | --- | --- | --- | --- | --- | --- | --- |
| Period | (1) | (2) | (3) | (4) | (5) | (6) | (7) | (8) | (9) | (10) | (11) | (12) |
|  | Absolute change (kg) | Average annual absolute change (kg) | Relative change (%) | Average annual relative change (%) | Absolute change | Average annual absolute change | Relative change (%) | Average annual relative change (%) | Absolute change (pp) | Average annual absolute change (pp) | Relative change (%) | Average annual relative change (%) |
| Early Adolescence (10-14) | 13,09 | 3,27 | 45,64 | 9,89 | 0,39 | 0,10 | 35,15 | 8,95 | -12,59 | -3,15 | -55,94 | -14,94 |
| Late Adolescence (15-19) | 2,95 | 0,95 | 6,77 | 2,18 | 0,16 | 0,04 | 23,44 | 5,39 | -3,16 | -0,99 | -38,77 | -9,78 |
